# A comparative analysis of the cholesterol–high-density lipoprotein–glucose index and the triglyceride–glucose index in predicting in-hospital mortality in critically ill ischemic stroke patients

**DOI:** 10.3389/fneur.2025.1664891

**Published:** 2025-10-22

**Authors:** Huang Luwen, Li Linlin, Yu Ming, Xu Lei

**Affiliations:** Department of Neurology, Suining Central Hospital, Suining, Sichuan, China

**Keywords:** ischemic stroke, cholesterol–high-density lipoprotein–glucose index, triglyceride–glucose index, mortality, eICU

## Abstract

**Background:**

The Cholesterol, high-density lipoprotein, and glucose (CHG) index has emerged as a potential indicator of metabolic disturbance, but its prognostic value in patients with ischemic stroke (IS) remains unclear. This study aimed to assess whether the CHG index could predict 28-day in-hospital mortality in critically ill IS patients and to compare its performance with the established triglyceride–glucose (TyG) index.

**Methods:**

We conducted a cohort analysis using data from the eICU database, involving 1,670 critically ill patients diagnosed with IS between 2014 and 2015. CHG and TyG indices were computed for each patient. Their associations with 28-day in-hospital mortality were examined using multivariable Cox regression. To further investigate the associations, restricted cubic spline (RCS) analysis was conducted. Kaplan–Meier curves were used to compare outcomes across different TyG and CHG groups. Predictive accuracy was compared using receiver operating characteristic (ROC) analysis. Subgroup analyses were performed to assess consistency across different clinical characteristics.

**Results:**

Among the study population, 158 (9.46%) patients died within 28 days of hospitalization. The CHG index showed a greater association with mortality (HR 1.554; 95% CI 1.198–2.018; *p* < 0.001) compared to the TyG index (HR 1.436; 95% CI 1.175–1.755; *p* < 0.001) in unadjusted models, and both remained significant after adjustment. RCS analysis demonstrated a linear relationship between both indices and 28-day in-hospital mortality. ROC curves showed similar discriminatory ability for the CHG and TyG indices. No significant interactions were observed in subgroup analyses (*p* > 0.05; *p* for interaction >0.05).

**Conclusions:**

Higher CHG index values are independently associated with increased 28-day mortality in critically ill IS patients, showing a linear relationship and predictive performance comparable to that of the TyG index.

## 1 Introduction

Stroke remains one of the leading causes of mortality worldwide, with ischemic stroke (IS) being a primary contributor (1–5). Critically ill IS patients often experience increased short-term mortality (6), making the early identification of prognostic factors crucial for effective clinical management. While individual biomarkers such as blood pressure (7), blood glucose (8), total cholesterol (TC) (9, 10), triglyceride (TG) (11), and C-reactive protein (12, 13) are associated with poor stroke outcomes, a single biomarker may not fully capture the complexity of the disease. Therefore, integrating multiple biomarkers provides a more comprehensive assessment, offering better predictive accuracy for patient prognosis. Therefore, identifying reliable biomarkers to predict mortality risk is essential for improving outcomes in these patients.

The unfavorable prognosis of IS is significantly influenced by insulin resistance (IR) (14–16). The triglyceride–glucose (TyG) index, a surrogate marker of IR, has been widely used in clinical practice (17, 18). Numerous studies have shown that the TyG index is closely associated with poor stroke outcomes, including patient mortality (19, 20). However, recent studies have highlighted a novel biomarker, the cholesterol, high-density lipoprotein, and glucose (CHG) index, which can be used to assess the risk of cardiovascular disease (21). Additionally, the CHG index has shown superior diagnostic efficiency for type 2 diabetes mellitus (DM) compared with the TyG index (22). However, to date, no studies have evaluated the predictive role of the CHG index for IS mortality. Both the TyG and CHG indices are composite biomarkers that integrate lipid and glucose levels and are associated with IR. This study aims to compare the CHG index with the TyG index to assess whether the CHG index can serve as a biomarker for predicting IS mortality and to evaluate its ability to predict IS mortality.

This study is based on the eICU database and aims to contribute to the development of predictive factors for short-term mortality in IS patients. This study will also deepen our understanding of the role of metabolic factors in IS outcomes, address existing gaps in the literature and provide more accurate biomarker references for clinical application.

## 2 Methods

### 2.1 Source of data

This study utilized data from the eICU Collaborative Research Database (eICU-CRD, version 2.0), a publicly available, multicenter ICU database developed by the Massachusetts Institute of Technology in collaboration with the eICU Research Institute. The database contains deidentified clinical data from over 200,000 ICU admissions across 208 U.S. hospitals between 2014 and 2015, including demographics, diagnoses, laboratory tests, medications, vital signs, and outcomes. Access was granted following completion of the CITI program and approval of the Data Use Agreement. The author Huang Luwen obtained the necessary authorization to access the dataset. All analyses adhered to HIPAA regulations and the Declaration of Helsinki. The database is available via PhysioNet (https://eicu-crd.mit.edu).

### 2.2 Study population

**Figure 1:**
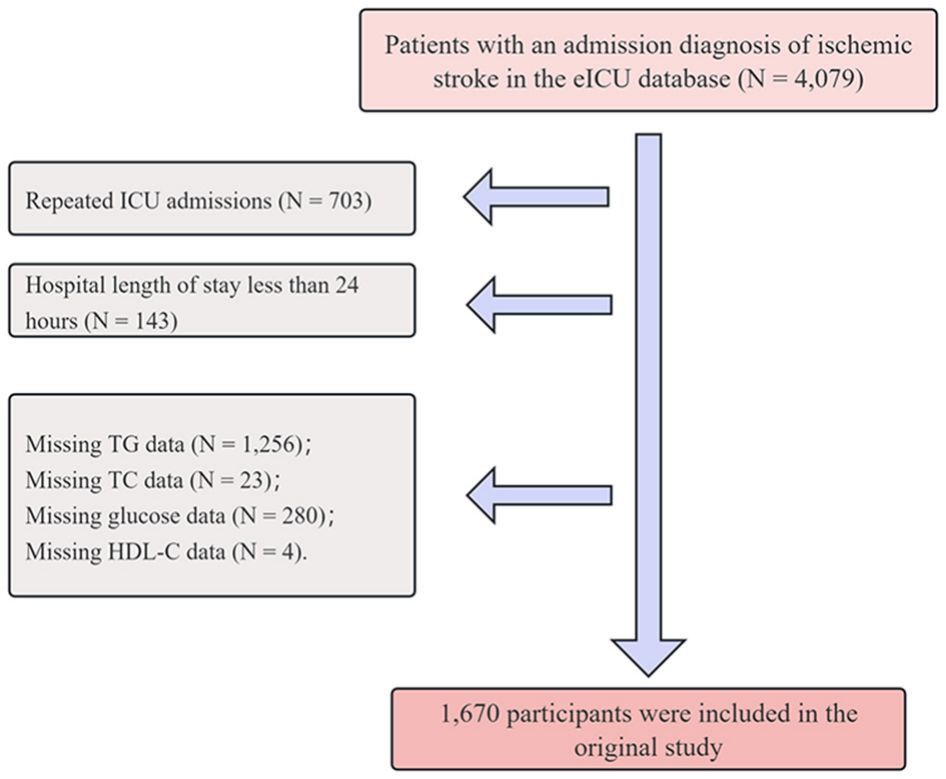
Flow chart of the study population.

A total of 4,079 patients diagnosed with IS were initially identified from the eICU database via the search terms “neurologic,” “disorders of vasculature,” “stroke,” and “ischemic stroke,” where the latter term is a subset of the broader categories mentioned (23). To ensure data quality and analytical validity, the following exclusion criteria were applied: (1) repeated ICU admissions (*N* = 703); (2) hospital length of stay less than 24 h (*N* = 143); (3) missing TG data (*N* = 1,256); (4) missing TC data (*N* = 23); (5) missing glucose data (*N* = 280); and (6) missing high-density lipoprotein cholesterol (HDL-C) data (*N* = 4). After these exclusions were applied, a total of 1,670 patients were included in the study. A detailed flowchart is illustrated in Figure 1.

### 2.3 Exposure and outcome definitions

The exposure variables, TyG and CHG indices, were calculated via the following standard formulas:

- TyG = ln [TG (mg/dl) × FBG (mg/dl)/2] (17);
- CHG = ln [TC (mg/dl) × FBG (mg/dl)/2 × HDL-C (mg/dl)] (21).

The primary outcome was 28-day all-cause in-hospital mortality.

### 2.4 Assessments of covariates

The variables extracted from the database included the following: sex, age, ethnicity, and body mass index (BMI); mechanical ventilation use and sequential organ failure assessment (SOFA) score; comorbidities including sepsis, chronic obstructive pulmonary disease (COPD), congestive heart failure (CHF), acute myocardial infarction (AMI), DM, pneumonia, and arrhythmia; and laboratory parameters such as glucose, blood urea nitrogen (BUN), serum creatinine, TC, TG, LDL-C, HDL-C, serum potassium, and serum sodium.

### 2.5 Missing variables

The distribution of variables with missing data is summarized in Supplementary Table S1, with most variables exhibiting a low proportion of missing values. To address this, multiple imputation using chained equations was employed under the assumption of missing data at random. Five imputed datasets were generated, and the results were pooled according to Rubin's rules to account for variability between imputations. This approach allowed for the preservation of the full cohort size and improved the accuracy and robustness of the statistical estimates.

### 2.6 Statistical analysis

Baseline characteristics are presented as the means ± standard deviations (SDs) or medians with interquartile ranges (IQRs) for continuous variables and as frequencies with percentages for categorical variables. Student's *t* test and one-way ANOVA were used for normally distributed continuous variables. Categorical variables were analyzed via either Fisher's exact test or the chi-square test and are reported as numbers and percentages.

To assess potential collinearity between the CHG and TyG indices and other covariates, we evaluated the generalized variance inflation factor (GVIF) and adjusted GVIF (Supplementary Tables S2, S3). The proportional hazards assumption was tested for all variables using the Schoenfeld residuals test. The associations between CHG, TyG, and 28-day in-hospital mortality were examined via both univariate and multivariate Cox proportional hazards models. Model 1 was unadjusted; Model 2 was adjusted for age, sex, and ethnicity; and Model 3 was further adjusted for mechanical ventilation use, SOFA score, DM, sepsis, COPD, CHF, AMI, arrhythmia, pneumonia, serum creatinine, BUN, serum potassium, and serum sodium.

Kaplan–Meier survival curves were generated to visualize the cumulative incidence of 28-day in-hospital mortality across different CHG and TyG categories. In addition, restricted cubic spline (RCS) models were used to explore the potential dose–response relationships between CHG, TyG, and 28-day mortality. To compare the predictive performance of CHG and TyG for short-term mortality, receiver operating characteristic (ROC) curve analysis was performed.

To further validate the robustness of our findings, several sensitivity analyses were conducted. First, Cox regression was repeated using a complete-case dataset after all missing values were excluded. Second, the analysis was repeated using multiple imputation to account for missing data. Third, a propensity score-weighted analysis was conducted to evaluate the association between CHG, TyG, and 28-day in-hospital mortality, with both CHG and TyG categorized into three groups: Q1, Q2, and Q3. Various weighting methods, including IPTW, overlap weighting, matching weighting, entropy weighting, and treated weighting, were applied to adjust for potential confounders. Forth, stratified analyses were performed to examine potential effect modifications across subgroups defined by age, sex, ethnicity, DM, arrhythmia, pneumonia, serum creatinine, BUN, serum potassium, and serum sodium.

All analyses were conducted via R software (version 3.3.2, The R Foundation, https://www.R-project.org) and Free Statistics software (version 1.7). A two-tailed *p* value < 0.05 was considered statistically significant.

## 3 Results

### 3.1 Baseline characteristics

The baseline characteristics of the study population (*n* = 1,670) were analyzed according to tertiles of the triglyceride–glucose index (TyG; Supplementary Table S4) and cholesterol–glucose index (CHG; Supplementary Table S5), defined as Q1, Q2, and Q3. Compared with those in Q1, patients in Q2 and Q3 were younger, had a lower proportion of males, and presented significantly higher BMIs, SOFA scores, and rates of mechanical ventilation (all *p* < 0.05). Laboratory parameters, including blood glucose, BUN, Scr, TC, TG, and LDL-C, were significantly increased in Q3, whereas HDL-C levels were significantly decreased (all *p* < 0.001). In addition, the prevalence of DM, pneumonia, and AMI was significantly greater in Q3 than in Q1 (all *p* < 0.05). The 28-day in-hospital mortality also increased significantly across TyG tertiles (Q1: 5.92%, Q2: 9.53%, and Q3: 12.93%; *p* < 0.001) and CHG tertiles (Q1: 5.75%, Q2: 9.35%, and Q3: 13.29%; *p* < 0.001). Compared with survivors, non-survivors had significantly higher SOFA scores, more frequent mechanical ventilation use, and elevated levels of glucose, BUN, and serum creatinine (all *p* < 0.001; Table 1).

**Table 1:** Baseline characteristics of participants classified by survival.

**Variables**	**Total (*N* = 1,670)**	**28-day survivors (*N* = 1,512)**	**28-day non-survivors (*N* =158)**	***P* value**
Age, mean (SD), year	70.69 ± 15.24	69.17 ± 13.87	65.55 ± 12.72	< 0.001
Gender (male), *n* (%)	276 (49.55)	267 (48.02)	258 (46.32)	< 0.001
**Ethnicity**, ***n*** **(%)**				
Caucasian	415 (74.77)	427 (76.94)	420 (75.68)	0.07
African American	75 (13.51)	70 (12.61)	53 (9.55)	
Other or unknown	65 (11.71)	58 (10.45)	82 (14.77)	
Body mass index, mean (SD), kg/m^2^	26.67 ± 6.49	29.00 ± 7.21	30.38 ± 6.83	< 0.001
Mechanical ventilation use, *n* (%)	67 (12.16)	90 (16.36)	133 (24.05)	< 0.001
SOFA score	2.00 (1.00, 3.00)	2.00 (1.00, 3.00)	2.00 (1.00, 4.00)	0.034
SEPSIS, *n* (%)	2 (0.36)	4 (0.72)	9 (1.62)	0.087
COPD, *n* (%)	23 (4.13)	21 (3.78)	16 (2.87)	0.509
CHF, *n* (%)	18 (3.23)	20 (3.6)	25 (4.49)	0.527
AMI, *n* (%)	4 (0.72)	12 (2.16)	22 (3.95)	0.001
Diabetes mellitus, *n* (%)	46 (8.35)	81 (14.73)	190 (34.36)	< 0.001
Pneumonia, *n* (%)	15 (2.69)	30 (5.4)	33 (5.92)	0.023
Arrhythmia, *n* (%)	102 (18.31)	102 (18.35)	75 (13.46)	0.043
Glucose, mean (SD), mg/dl	106.23 ± 23.43	122.43 ± 31.84	172.34 ± 74.73	< 0.001
BUN, median (IQR), mg/dl	18.17 ± 12.42	18.27 ± 10.82	21.11 ± 14.51	< 0.001
Serum creatinine, median (IQR), mg/dl	0.83 (0.69, 1.05)	0.90 (0.73, 1.12)	0.92 (0.76, 1.29)	< 0.001
TC, median (IQR), mg/dl	145.50 ± 37.49	157.59 ± 40.53	174.83 ± 53.62	< 0.001
TG, median (IQR), mg/dl	68.00 (55.00, 84.00)	110.00 (91.00, 128.25)	176.00 (138.00, 238.00)	< 0.001
LDL-C, mean (SD), mg/dl	81.72 ± 31.88	92.08 ± 36.37	96.16 ± 44.71	< 0.001
HDL-C, mean (SD), mg/dl	50.29 ± 16.27	43.18 ± 13.82	38.23 ± 13.33	< 0.001
Serum potassium, mean (SD), mmol/L	3.96 ± 0.50	3.93 ± 0.53	3.98 ± 0.57	0.261
Serum sodium, mean (SD), mmol/L	139.00 ± 3.99	139.31 ± 3.35	138.99 ± 3.84	0.278
CHG, mean (SD)	8.15 ± 0.27	8.76 ± 0.16	9.59 ± 0.49	< 0.001
TyG, mean (SD)	33 (5.92)	53 (9.53)	72 (12.93)	< 0.001

SOFA, sequential organ failure assessment; DM, diabetes mellitus; COPD, chronic obstructive pulmonary disease; CHF, congestive heart failure; AMI, acute myocardial infarction; BUN, blood urea nitrogen; TC, total cholesterol; TG, triglycerides; LDL-C, low-density lipoprotein cholesterol; HDL-C, high-density lipoprotein cholesterol; CHG, cholesterol, high-density lipoprotein, and glucose index; TyG, triglyceride–glucose; SD, standard deviation; IQR, interquartile range.

### 3.2 Associations of CHG and TyG with 28-day in-hospital mortality

According to the univariate Cox regression analysis, both the CHG and TyG indices were significantly associated with 28-day in-hospital mortality (Supplementary Table S6). The proportional hazards assumption was tested using the Schoenfeld residuals test, and all variables had *p*-values greater than 0.05. Compared with those in the lowest CHG group (Q1), patients in Q3 presented a significantly increased risk of death (HR = 1.707, 95% CI: 1.125–2.592, *p* = 0.012). Similarly, individuals in the highest TyG group (Q3) had a greater mortality risk relative than those in Q1 did (HR = 1.832, 95% CI: 1.212–2.770, *p* = 0.004). These associations remained robust after adjusting for potential confounding factors (Table 2). In the fully adjusted model (Model 3), CHG as a continuous variable remained independently associated with 28-day mortality (HR = 1.601, 95% CI: 1.184–2.164, *p* = 0.002), and Q3 maintained statistical significance (HR = 1.758, 95% CI: 1.120–2.759, *p* = 0.014). Similarly, the TyG index as a continuous variable was independently associated with 28-day mortality in the fully adjusted model (HR = 1.433, 95% CI: 1.118–1.836, *p* = 0.005), although the association for the highest TyG quartile (Q3) did not reach statistical significance (*p* = 0.054). These findings suggest a more robust and consistent association for the CHG index in predicting short-term mortality than the TyG index does.

**Table 2:** Cox regression models for the association between the CHG index, TyG index, and 28-day in-hospital mortality.

**Categories**	**Event, (*n* %)**	**Model 1**		**Model 2**		**Model 3**	
		**HR (95% CI)**	***P*** **value**	**HR (95% CI)**	***P*** **value**	**HR (95% CI)**	***P*** **value**
**CHG index**							
Continuous	158 (9.5)	1.554 (1.198–2.018)	< 0.001	1.778 (1.351–2.34)	< 0.001	1.601 (1.184–2.164)	0.002
**Quartile**							
Q1	32 (5.7)	1(Ref)		1(Ref)		1(Ref)	
Q2	52 (9.4)	1.446 (0.93–2.247)	0.101	1.513 (0.971–2.357)	0.067	1.425 (0.901–2.253)	0.130
Q3	74 (13.3)	1.707 (1.125–2.592)	0.012	1.938 (1.266–2.967)	0.002	1.758 (1.12–2.759)	0.014
*P* for trend			0.013		0.002		0.014
**TyG index**							
Continuous	158 (9.5)	1.436 (1.175–1.755)	< 0.001	1.648 (1.328–2.047)	< 0.001	1.433 (1.118–1.836)	0.005
**Quartile**							
Q1	33 (5.9)	1(Ref)		1(Ref)		1(Ref)	
Q2	53 (9.5)	1.49 (0.964–2.302)	0.072	1.536 (0.993–2.375)	0.054	1.469 (0.942–2.291)	0.090
Q3	72 (12.9)	1.832 (1.212–2.77)	0.004	2.111 (1.382–3.225)	< 0.001	1.564 (0.993–2.465)	0.054
*P* for trend			0.004		< 0.001		0.063

Model 1: unadjusted.

Model 2: adjusted for age, gender, and ethnicity.

Model 3: adjusted for Model 2 plus, ventilation status, SOFA score, diabetes, sepsis, COPD, CHF, AMI, arrhythmia, pneumonia, serum creatinine, BUN, serum potassium, and sodium levels.

CHG, cholesterol, high-density lipoprotein, and glucose index; TyG, triglyceride–glucose.

**Figure 2:**
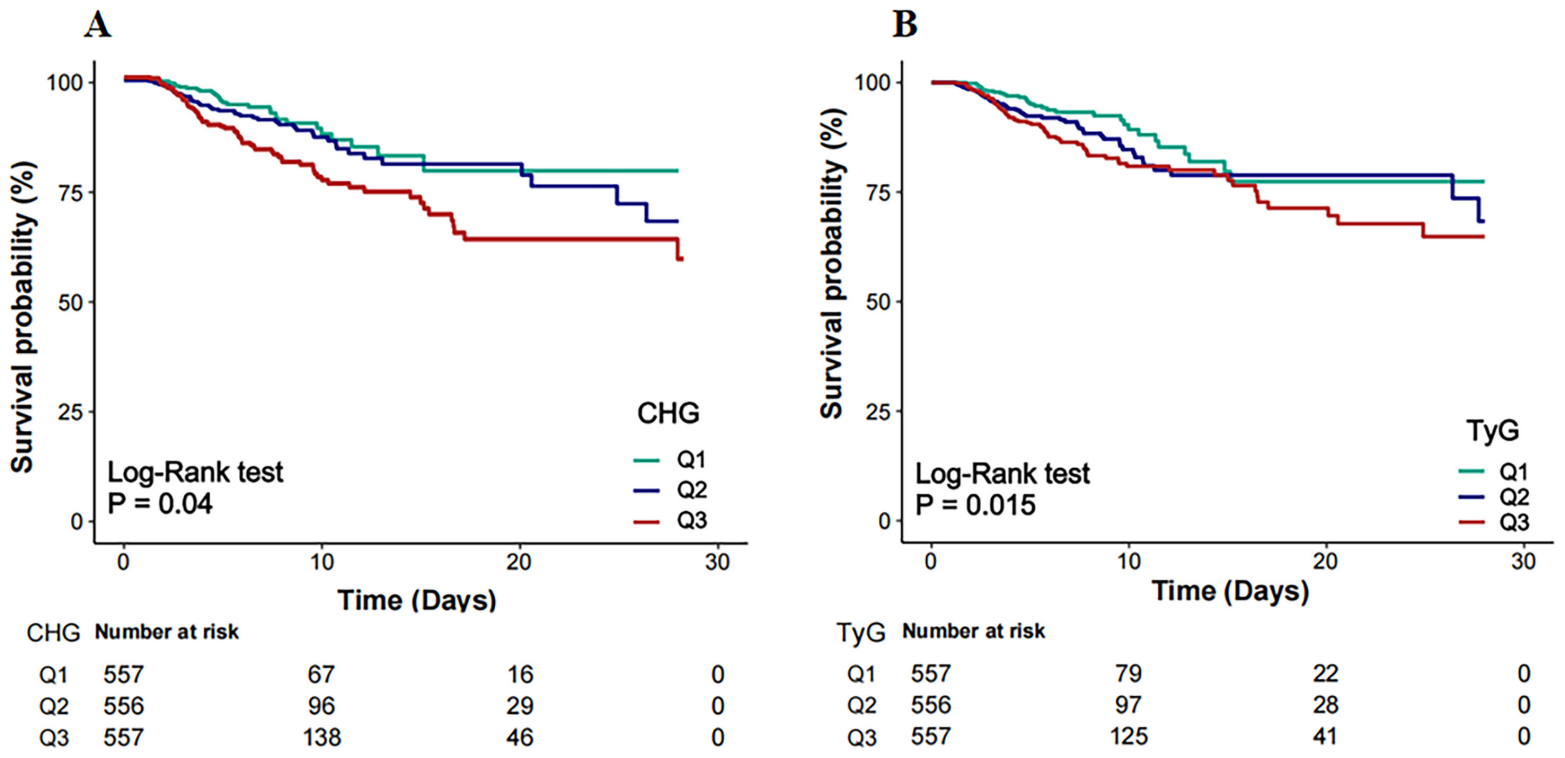
Kaplan–Meier survival curves for 28-day in-hospital mortality by CHG **(A)** and TyG **(B)**. CHG, cholesterol, high-density lipoprotein, and glucose index; TyG, triglyceride–glucose.

Kaplan–Meier survival analysis revealed that the both CHG and TyG indices were significantly associated with 28-day survival outcomes (Figures 2A, B). Patients in Q3 of CHG and TyG presented significantly lower survival probabilities than those in the Q1 did, as shown by the log-rank test (*p* = 0.04 and *p* = 0.015, respectively).

### 3.3 Dose–response associations between CHG/TyG and 28-day in-hospital mortality

**Figure 3:**
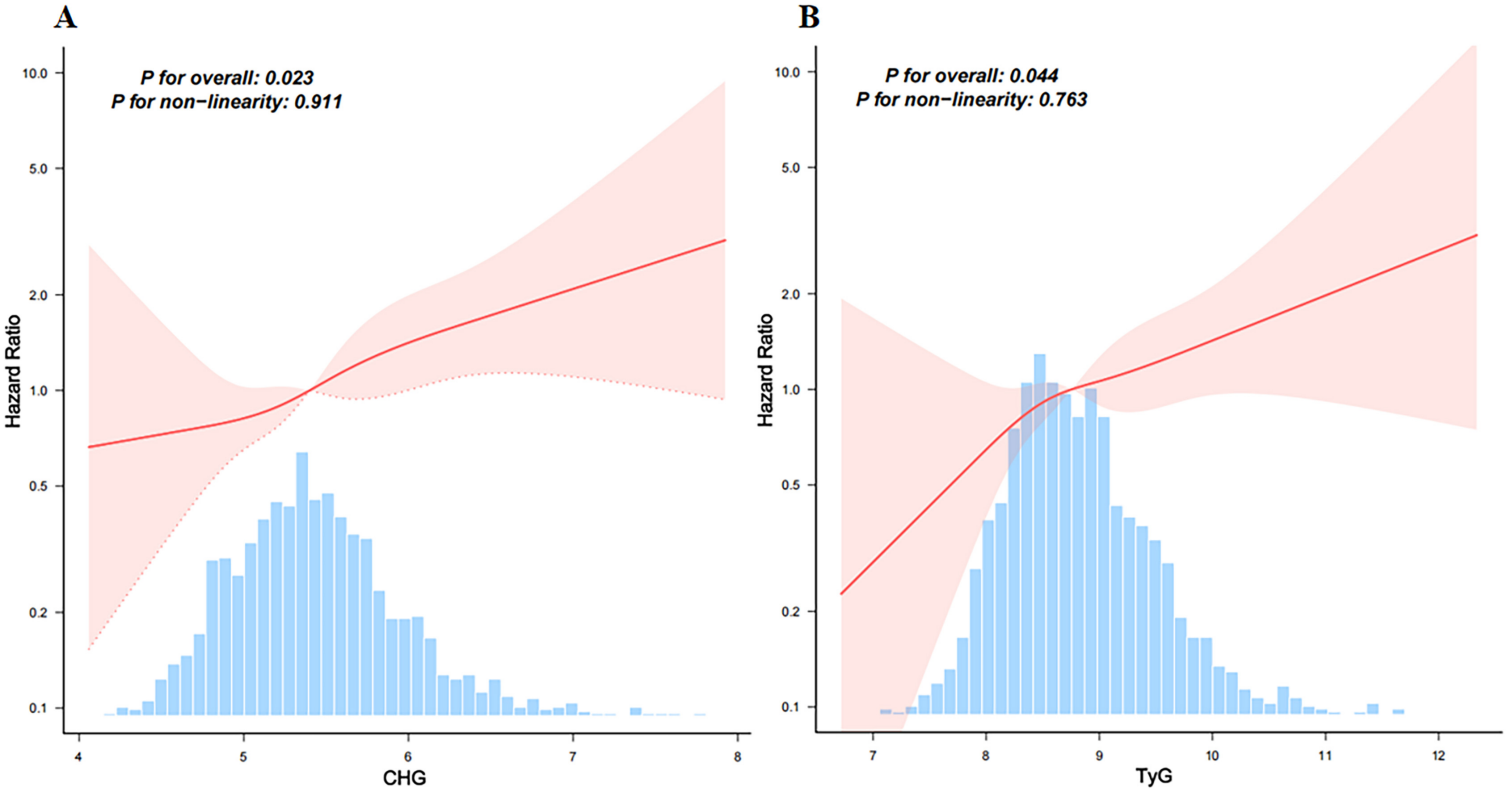
Restricted cubic spline models analyzed the relationship between CHG **(A)** and TyG **(B)**, and 28-day hospital mortality. Adjusted for age, gender, ethnicity, ventilation status, SOFA score, diabetes, sepsis, COPD, CHF, AMI, arrhythmia, pneumonia, serum creatinine, BUN, serum potassium, and sodium levels. CHG, cholesterol, high-density lipoprotein, and glucose index; TyG, triglyceride–glucose.

RCS regression was performed to explore the dose–response relationships between the CHG and TyG indices and 28-day in-hospital mortality (Figures 3A, B). Both CHG (*p* for overall = 0.023) and TyG (*p* for overall = 0.044) were significantly associated with increased risk of mortality. No evidence of non-linear was observed (*p* for non-linear = 0.911 for CHG; *p* for non-linear = 0.763 for TyG), indicating that the relationships between these indices and mortality risk are linear.

### 3.4 Comparison of CHG and TyG for 28-day in-hospital mortality discrimination

**Figure 4:**
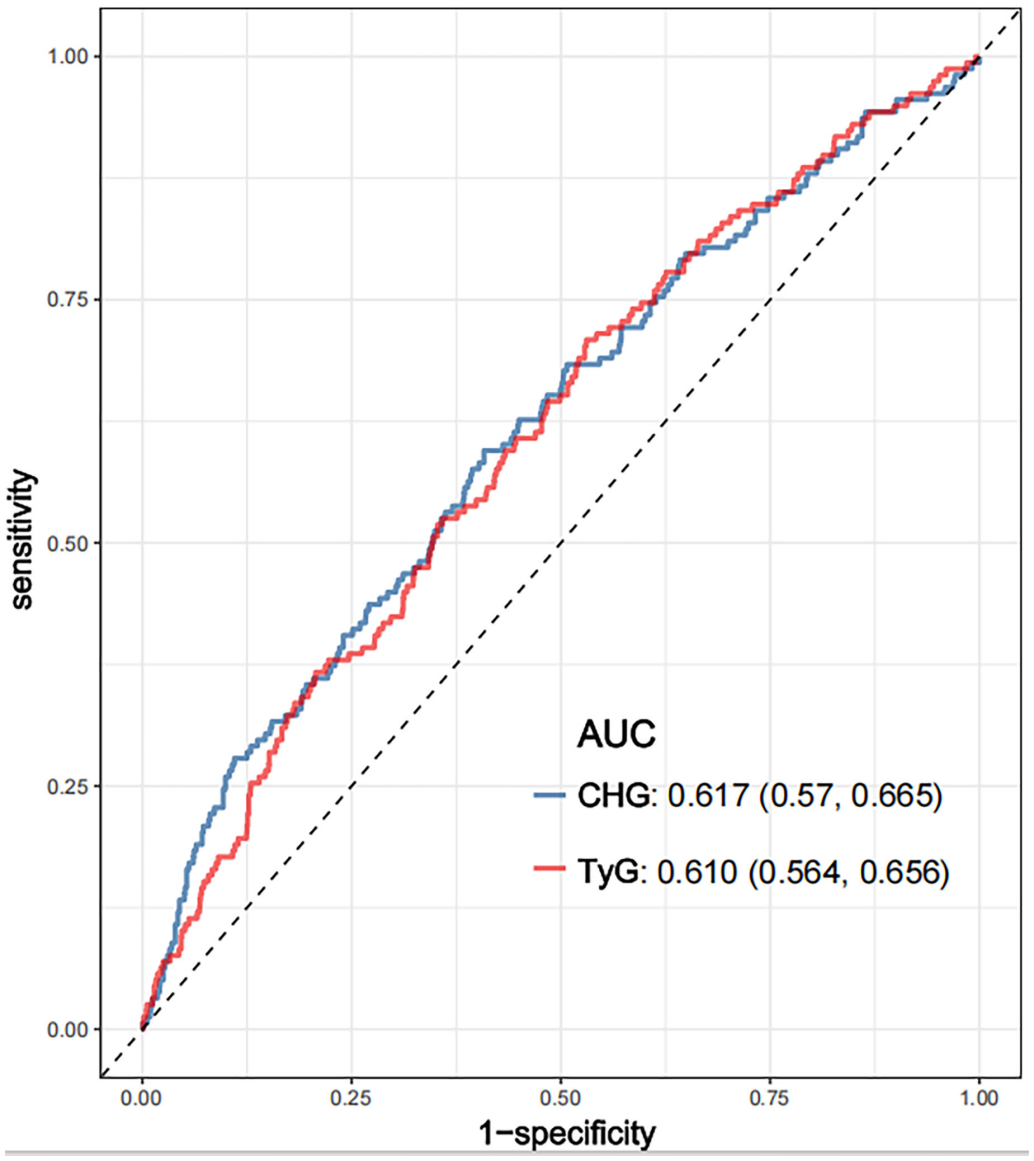
ROC curves compared the predictive efficacy of the CHG and TyG for 28-day hospital mortality. CHG: cholesterol, high-density lipoprotein, and glucose index; TyG, triglyceride–glucose; ROC, receiver operating characteristic.

ROC analysis was conducted to evaluate the ability of the CHG and TyG indices to predict 28-day hospital mortality (Figure 4). The CHG index demonstrated an area under the curve (AUC) of 0.617 (95% CI: 0.570–0.665), which was slightly greater than that of the TyG index (AUC = 0.610; 95% CI: 0.564–0.656), indicating that both indices have comparable predictive abilities for short-term mortality risk.

### 3.5 Subgroup analysis of the CHG and TyG indices

**Figure 5:**
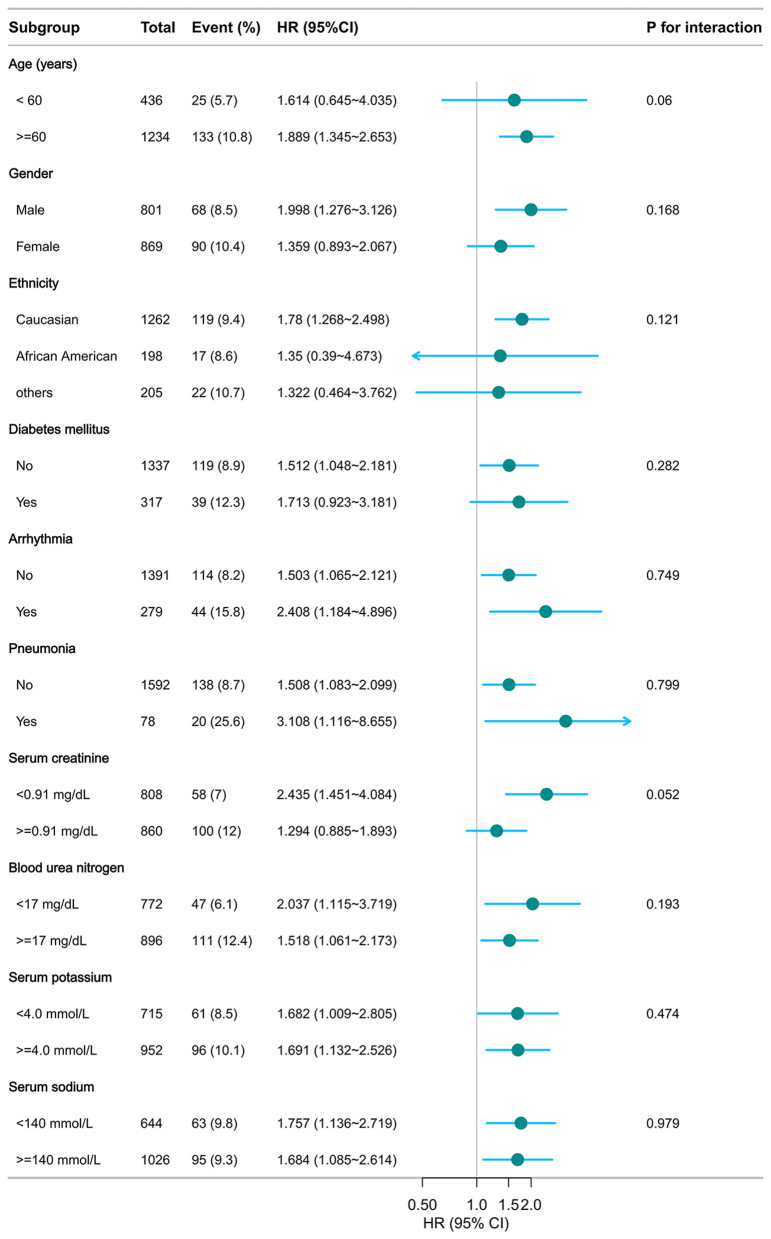
Subgroup analysis of CHG in predicting 28-day hospital mortality. Adjusted for age, gender, ethnicity, ventilation status, SOFA score, diabetes, sepsis, COPD, CHF, AMI, arrhythmia, pneumonia, serum creatinine, BUN, serum potassium, and sodium levels. CHG: cholesterol, high-density lipoprotein, and glucose index.

**Figure 6:**
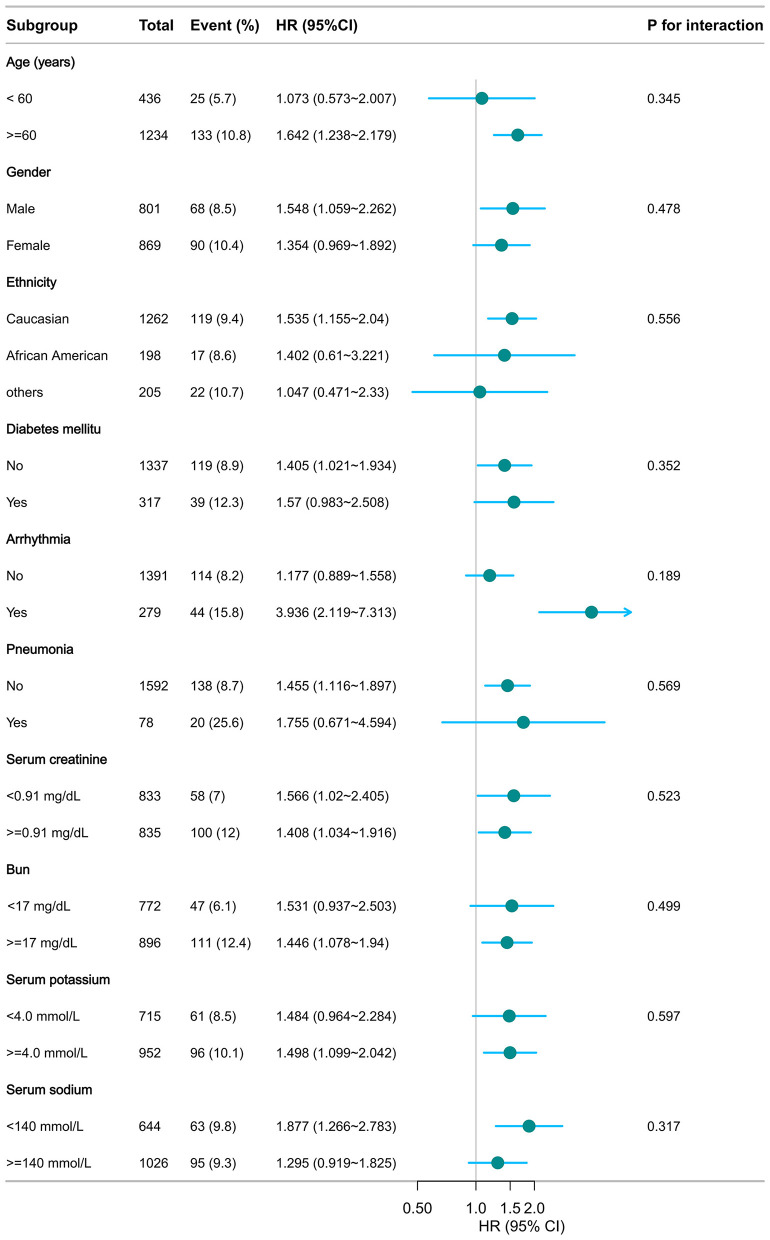
Subgroup analysis of TyG in predicting 28-day hospital mortality. Adjusted for age, gender, ethnicity, ventilation status, SOFA score, diabetes, sepsis, COPD, CHF, AMI, arrhythmia, pneumonia, serum creatinine, BUN, serum potassium, and sodium levels. TyG, triglyceride–glucose.

As illustrated in Figures 5, 6, no significant interactions were observed between CHG or TyG indices and clinical subgroups, including age, sex, mechanical ventilation status, serum creatinine, BUN, or the presence of pneumonia (all *p* for interaction >0.05). These findings suggest that the associations of both the CHG and TyG indices with 28-day in-hospital mortality were consistent across different baseline characteristics and were not significantly modified by stratifying variables.

### 3.6 Sensitivity analyses

**Figure 7:**
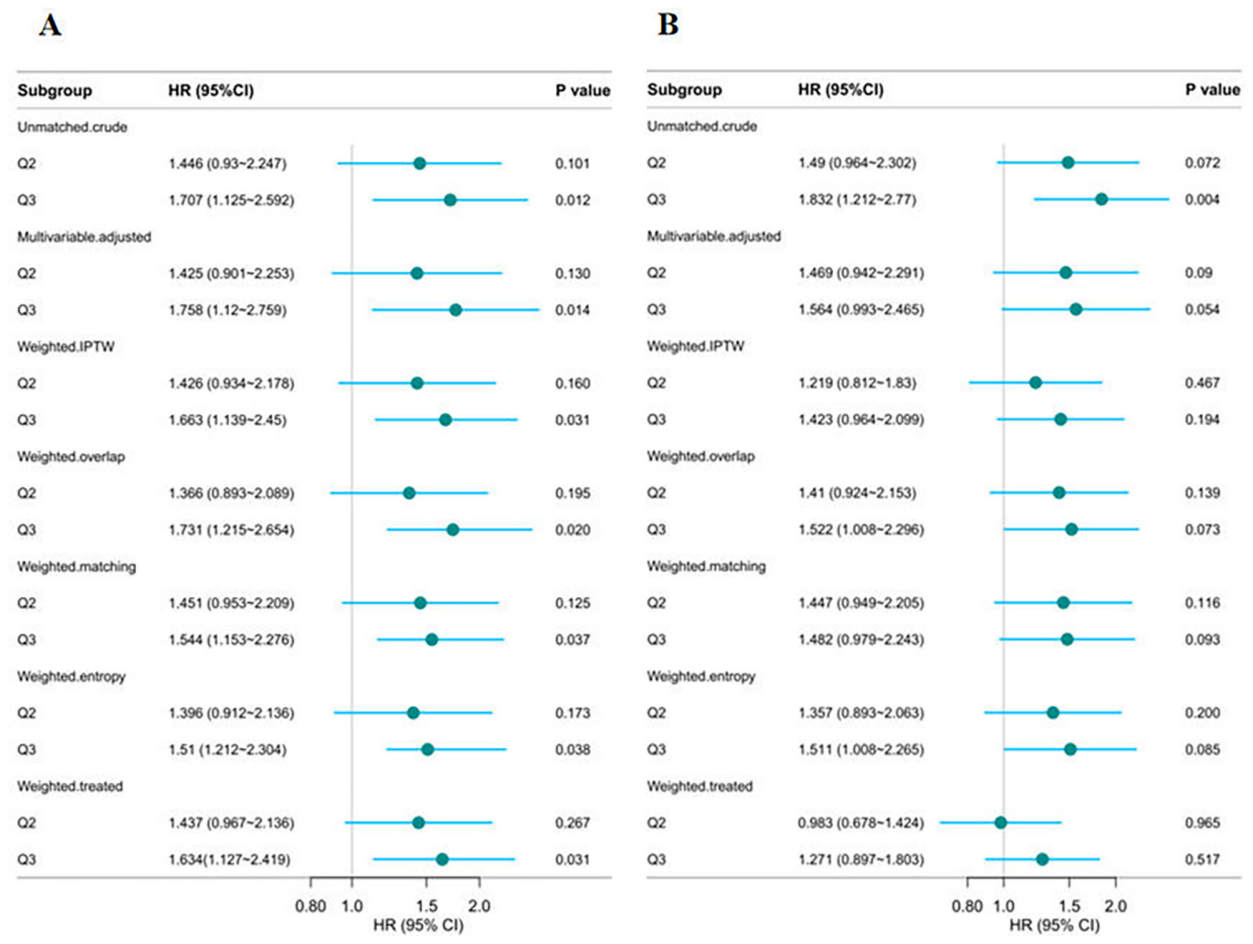
Multi-group propensity score weighting analysis of the relationship between CHG **(A)** and TyG **(B)**, and 28-day hospital mortality. Adjusted for age, gender, ethnicity, ventilation status, SOFA score, diabetes, sepsis, COPD, CHF, AMI, arrhythmia, pneumonia, serum creatinine, BUN, serum potassium, and sodium levels. CHG, cholesterol, high-density lipoprotein, and glucose index; TyG, triglyceride–glucose.

To assess the robustness of the findings, several sensitivity analyses were performed. First, a complete-case analysis excluding individuals with missing data demonstrated that the CHG index remained significantly associated with 28-day in-hospital mortality, both as a continuous variable (HR = 1.601, 95% CI: 1.184–2.164, *p* = 0.002) and in Q3 (Q3 vs. Q1: HR = 1.758, 95% CI: 1.120–2.759, *p* = 0.014; Supplementary Table S7). The TyG index also showed a significant association in the continuous model (HR = 1.433, 95% CI: 1.118–1.836, *p* = 0.005) but not in the categorical model (Q3 vs. Q1: *p* = 0.090). Second, multiple imputation yielded consistent results (Supplementary Table S8). CHG remained strongly associated with mortality in both the continuous (HR = 1.559, 95% CI: 1.156–2.104, *p* = 0.004) and categorical models (Q3 vs. Q1: HR = 1.728, 95% CI: 1.102–2.710, *p* = 0.017). TyG also remained a significant as a continuous variable (*p* = 0.007), whereas its categorical association with Q3 remained non-significant (*p* = 0.058). Third, in the propensity score-weighted analysis, the three-group classification of CHG demonstrated a significant association with 28-day in-hospital mortality (Figure 7A). Regardless of the weighting method used (IPTW, overlap weighting, matching weighting, entropy weighting, or treated weighting), both the Q3 groups were significantly associated with an increased risk of mortality. However, the relationship between TyG and 28-day in-hospital mortality was not stable (Figure 7B). Forth, E-value analysis revealed that an unmeasured confounder would need to be associated with both the exposure and outcome with a risk ratio of at least 2.91 for CHG and 2.50 for TyG to fully account for the observed associations, indicating moderate robustness to potential unmeasured confounding (Supplementary Figure S1).

## 4 Discussion

This study, which is based on the eICU multicenter critical care database, is the first to examine the predictive value of the CHG index for 28-day in-hospital mortality in critically ill IS patients and to compare it with the established TyG index. These findings indicate that, after extensive adjustment for various confounding factors, both indices are significantly associated with an increased risk of 28-day in-hospital mortality. RCS analysis further demonstrated a positive linear relationship with 28-day in-hospital mortality. Notably, the CHG index demonstrated a higher HR than the TyG index did, and ROC analysis further revealed that the CHG index had a stronger ability to predict 28-day in-hospital mortality than did the TyG index.

The TyG index, a well-established marker for IR, has been associated with adverse outcomes in stroke patients (24, 25). Elevated TyG is associated with unfavorable functional outcomes and an increased risk of recurrent stroke (26, 27). Studies using the MIMIC-IV and eICU databases have shown a significant correlation between elevated TyG levels and mortality following both ischemic and hemorrhagic stroke (28, 29), supporting its role as a prognostic tool. Additionally, TyG-derived indices, such as TyG-BMI (30) and TyG-WC (31), are strongly associated with mortality after stroke. However, the study by Bukke et al. revealed no significant associations between TyG and IS recurrence or mortality (32), likely because of the a small sample size. Despite this, most studies suggest a positive relationship between the TyG index and mortality in IS patients.

This study is the first to explore the association between the CHG index and 28-day in-hospital mortality in critically ill IS patients and to compare it with the TyG index. In our study, ROC curve analysis suggested that CHG and TyG have similar predictive power for short-term mortality. However, after adjusting for confounding factors, the CHG index had a greater HR and more stable *p*-values than the TyG index did. This may indicate that, while their predictive power is similar, the CHG index offers a more sensitive measure, potentially owing to its more comprehensive evaluation of metabolic dimensions, which encompass a broader range of factors than TyG. As a novel biomarker introduced in 2024, the CHG index innovatively integrates three key metabolic parameters—TC, HDL-C, and glucose—enabling a comprehensive assessment of both metabolic function and vascular health (22). Specifically, elevated LDL-C levels increase the risk of IS mortality by promoting plaque formation, exacerbating vascular obstruction, and reducing brain tissue perfusion (33–35). In contrast, HDL-C exerts a protective effect by inhibiting cholesterol deposition and delaying the progression of atherosclerosis, thus reducing stroke-related mortality (36). In comparison, while the TyG index effectively reflects IR, its lack of consideration for critical vascular risk factors such as TC limits its predictive value for stroke outcomes.

From a pathophysiological perspective, the TyG index and CHG index influence stroke prognosis through different molecular pathways. The TyG index primarily reflects metabolic disorders associated with insulin resistance (37), including high blood glucose and elevated TG, which indirectly worsen cerebral blood flow dynamics by promoting atherosclerosis, damaging endothelial function, and increasing vascular stiffness, ultimately increasing stroke risk (38–41). In contrast, the CHG index, which integrates TC and glucose, provides a more comprehensive metabolic-vascular health evaluation system. Notably, large-scale epidemiological studies (*n* = 9,704) have confirmed that the CHG index outperforms the TyG index in the diagnosis of type 2 diabetes (22), indicating its prominent value in metabolic disease risk assessment. Moreover, Degang et al. demonstrated that the CHG index has a relatively high HR for cardiovascular disease risk stratification, further validating its clinical potential as a comprehensive predictive tool (21). Although these studies differ in terms of endpoints from the current study, they collectively reinforce the superior predictive value of the CHG index for metabolic-related diseases, providing a solid theoretical foundation for the conclusions of this study.

This study is the first to reveal a significant positive correlation between the CHG index and short-term mortality risk in critically ill IS patients while also comparing it to the widely recognized TyG index. Additionally, the study employed various statistical methods, including Cox regression, RCS, ROC curve, subgroup analysis, and multiple imputation, to develop a comprehensive assessment model. This multifaceted approach not only enhanced the diversity of the analysis but also strengthened the reliability and depth of the findings. Furthermore, the use of a large-scale cohort study based on the eICU database effectively minimized selection bias and ensured the representativeness of the sample. Finally, this study provides a new reference for clinicians assessing the risk of critically ill IS patients, facilitating personalized management and enabling targeted interventions for a broader population.

However, there are several limitations in this study. First, it relies on data from the eICU database, which, although representative, does not capture the characteristics of populations from other countries, limiting its external validity. Second, the lack of stroke subtype information in the database limits the ability to analyze and compare the predictive value of the two indices for different IS types. Third, although the study accounted for multiple confounding factors, it still lacked several important variables, such as the National Institutes of Health Stroke Scale score, hypertension, cause of death, and interventions including intravenous thrombolysis, thrombectomy, or pharmacological treatments. These limitations may impact the comprehensiveness of the results. In the future, we plan to collect more comprehensive data to facilitate further analysis. Fourth, the absence of long-term follow-up data restricts ability of these indices to predict long-term mortality risk in critically ill IS patients. Therefore, future research should conduct multicenter, large-sample, and long-term studies across diverse populations and various stroke subtypes to further explore the prognostic value of these two indices.

## 5 Conclusion

An elevated CHG index is significantly associated with increased 28-day in-hospital mortality in critically ill IS patients, indicating a linear relationship. CHG also demonstrates predictive performance similar to that of the TyG index in assessing mortality risk. However, its broader evaluation of metabolic factors may make it a superior marker. Integrating CHG into clinical practice could enhance decision-making and aid in the early identification of high-risk patients in critical care settings.

## Data Availability

The datasets presented in this study can be found in online repositories. The names of the repository/repositories and accession number(s) can be found below: the data utilized in this study were obtained from the eICU Collaborative Research Database, which is publicly available via PhysioNet (https://eicu-crd.mit.edu) following the completion of a data use agreement.
