# Supplementary material for: A comparative analysis of the cholesterol–high-density lipoprotein–glucose index and the triglyceride–glucose index in predicting in-hospital mortality in critically ill ischemic stroke patients
Source: Front Neurol. 2025 Oct 22;16:1664891. doi: 10.3389/fneur.2025.1664891 (PMC12587768; doi:10.3389/fneur.2025.1664891)
Supplement: Supplementary file 1 [file Table_1.docx]

| Table S1.  Distribution of variables with missing data. | | |
| --- | --- | --- |
| **Variables** | Number of Missing | **Missing proportion(%)** |
| Blood urea nitrogen | 2 | 0.12 |
| Diabetes mellitus | 16 | 0.96 |
| Ethnicity | 5 | 0.3 |
| Serum potassium | 3 | 0.18 |
| Serum creatinine | 2 | 0.12 |
| Mechanical ventilation use | 16 | 0.96 |

| Table S2.  Assessment of collinearity among independent variables in the final regression model (CHG). | | | | |
| --- | --- | --- | --- | --- |
| **Variables** | **GVIF** | **Df** | **Adjusted GVIF** | **Collinearity** |
| Age | 1.281 | 1 | 1.132 | No |
| Gender | 1.063 | 1 | 1.031 | No |
| Ethnicity | 1.182 | 2 | 1.043 | No |
| Mechanical ventilation use | 1.291 | 1 | 1.136 | No |
| SOFA score | 1.646 | 1 | 1.283 | No |
| DM | 1.164 | 1 | 1.079 | No |
| SEPSIS | 1.115 | 1 | 1.056 | No |
| COPD | 1.115 | 1 | 1.056 | No |
| CHF | 1.199 | 1 | 1.095 | No |
| AMI | 1.067 | 1 | 1.033 | No |
| Arrhythmia | 1.078 | 1 | 1.038 | No |
| Pneumonia | 1.11 | 1 | 1.054 | No |
| Serum creatinine | 2.32 | 1 | 1.523 | No |
| BUN | 2.711 | 1 | 1.647 | No |
| Serum potassium | 1.36 | 1 | 1.166 | No |
| Serum sodium | 1.278 | 1 | 1.13 | No |

SOFA, sequential organ failure assessment; DM, diabetes mellitus; COPD, chronic obstructive pulmonary disease; CHF, congestive heart failure; AMI, acute myocardial infarction; BUN, blood urea nitrogen; CHG: cholesterol, high-density lipoprotein, and glucose index.

| Table S3.  Assessment of collinearity among independent variables in the final regression model (TyG). | | | | |
| --- | --- | --- | --- | --- |
| **Variables** | **GVIF** | **Df** | **Adjusted GVIF** | **Collinearity** |
| Age | 1.288 | 1 | 1.135 | No |
| Gender | 1.083 | 1 | 1.041 | No |
| Ethnicity | 1.316 | 1 | 1.147 | No |
| Mechanical ventilation use | 1.655 | 1 | 1.286 | No |
| SOFA score | 1.237 | 1 | 1.112 | No |
| DM | 1.115 | 1 | 1.056 | No |
| SEPSIS | 1.101 | 1 | 1.049 | No |
| COPD | 1.183 | 1 | 1.088 | No |
| CHF | 1.071 | 1 | 1.035 | No |
| AMI | 1.081 | 1 | 1.04 | No |
| Arrhythmia | 1.097 | 1 | 1.047 | No |
| Pneumonia | 2.358 | 1 | 1.536 | No |
| Serum creatinine | 2.697 | 1 | 1.642 | No |
| BUN | 1.363 | 1 | 1.167 | No |
| Serum potassium | 1.233 | 1 | 1.11 | No |
| Serum sodium | 1.18 | 2 | 1.042 | No |

SOFA, sequential organ failure assessment; DM, diabetes mellitus; COPD, chronic obstructive pulmonary disease; CHF, congestive heart failure; AMI, acute myocardial infarction; BUN, blood urea nitrogen; TyG: triglyceride–glucose.

| Table S4. Baseline characteristics of participants classified by the cholesterol, high-density lipoprotein, and glucose (CHG). | | | | |
| --- | --- | --- | --- | --- |
| **Variables** | **Low CHG**  **(< 5.2)** | **Middle CHG**  **(5.2-5.6)** | **High CHG**  **(>5.6)** | **P value** |
| N | 557 | 556 | 557 |  |
| Age, mean (SD), year | 71.06 ± 14.28 | 69.06 ± 14.32 | 65.30 ± 13.21 | < 0.001 |
| Gender (male), n (%) | 325 (58.35) | 255 (45.86) | 221 (39.68) | < 0.001 |
| Ethnicity, n (%) |  |  |  | 0.188 |
| Caucasian | 432 (77.84) | 427 (76.94) | 403 (72.61) |  |
| African American | 60 (10.81) | 68 (12.25) | 70 (12.61) |  |
| Other or unknown | 63 (11.35) | 60 (10.81) | 82 (14.77) |  |
| Body mass index, mean (SD), kg/m^2^ | 26.80 ± 6.88 | 29.36 ± 7.31 | 29.90 ± 6.44 | < 0.001 |
| Mechanical ventilation use, n (%) | 81 (14.62) | 82 (14.91) | 127 (23.09) | < 0.001 |
| SOFA score | 2.00 (1.00, 3.00) | 2.00 (1.00, 3.00) | 2.00 (1.00, 4.00) | 0.021 |
| SEPSIS, n (%) | 2 (0.36) | 4 (0.72) | 9 (1.62) | 0.087 |
| COPD, n (%) | 29 (5.21) | 17 (3.06) | 14 (2.51) | 0.038 |
| CHF, n (%) | 20 (3.59) | 14 (2.52) | 29 (5.21) | 0.06 |
| AMI, n (%) | 7 (1.26) | 10 (1.8) | 21 (3.77) | 0.012 |
| DM, n (%) | 48 (8.66) | 93 (16.91) | 176 (32) | < 0.001 |
| Pneumonia, n (%) | 21 (3.77) | 19 (3.42) | 38 (6.82) | 0.012 |
| Arrhythmia, n (%) | 103 (18.49) | 90 (16.19) | 86 (15.44) | 0.363 |
| Glucose, mean (SD), mg/dL | 102.38 ± 19.01 | 122.70 ± 28.96 | 175.92 ± 73.72 | < 0.001 |
| BUN, median (IQR), mg/dL | 17.57 ± 11.83 | 19.25 ± 12.13 | 20.73 ± 13.98 | < 0.001 |
| Serum creatinine, median (IQR), mg/dl | 0.82 (0.67, 1.04) | 0.90 (0.74, 1.14) | 0.96 (0.76, 1.29) | < 0.001 |
| Total cholesterol, median (IQR), mg/dL | 141.16 ± 33.78 | 157.65 ± 37.98 | 179.11 ± 55.21 | < 0.001 |
| Triglycerides, median (IQR), mg/dL | 80.00  (61.00, 107.00) | 104.00  (79.00, 142.00) | 148.00  (106.00, 210.00) | < 0.001 |
| LDL-C, mean (SD), mg/dL | 71.11 ± 25.40 | 91.09 ± 31.56 | 107.27 ± 45.74 | < 0.001 |
| HDL-C, mean (SD), mg/dL | 53.29 ± 16.30 | 42.74 ± 12.27 | 35.68 ± 11.55 | < 0.001 |
| Serum, potassium, mean (SD), mmol/L | 3.94 ± 0.52 | 3.92 ± 0.49 | 4.01 ± 0.59 | 0.023 |
| Sodium, mean (SD), mmol/L | 139.35 ± 3.83 | 139.25 ± 3.45 | 138.70 ± 3.89 | 0.008 |
| CHG, mean (SD) | 4.91 ± 0.21 | 5.41 ± 0.12 | 6.03 ± 0.39 | < 0.001 |
| Hospital 28 mortality, n (%) | 32 (5.75) | 52 (9.35) | 74 (13.29) | < 0.001 |

SOFA, sequential organ failure assessment; DM, diabetes mellitus; COPD, chronic obstructive pulmonary disease; CHF, congestive heart failure; AMI, acute myocardial infarction; BUN, blood urea nitrogen; TC, total cholesterol; TG, triglycerides; LDL-C, low-density lipoprotein cholesterol; HDL-C, high-density lipoprotein cholesterol; CHG: cholesterol, high-density lipoprotein.

SD, standard deviation; IQR, interquartile range.

| Table S5. Baseline characteristics of participants classified by the triglyceride-glucose (TyG). | | | | |
| --- | --- | --- | --- | --- |
| **Variables** | **Low CHG**  **(< 5.2)** | **Middle CHG (5.2-5.6)** | **High CHG**  **(>5.6)** | **P value** |
| N | 70.69 ± 15.24 | 69.17 ± 13.87 | 65.55 ± 12.72 | < 0.001 |
| Age, mean (SD), year | 276 (49.55) | 267 (48.02) | 258 (46.32) | < 0.001 |
| Gender (male), n (%) |  |  |  | 0.07 |
| Ethnicity, n (%) | 415 (74.77) | 427 (76.94) | 420 (75.68) |  |
| Caucasian | 75 (13.51) | 70 (12.61) | 53 (9.55) |  |
| African American | 65 (11.71) | 58 (10.45) | 82 (14.77) |  |
| Other or unknown | 26.67 ± 6.49 | 29.00 ± 7.21 | 30.38 ± 6.83 | < 0.001 |
| Body mass index, mean (SD), kg/m^2^ | 67 (12.16) | 90 (16.36) | 133 (24.05) | < 0.001 |
| Mechanical ventilation use, n (%) | 2.00 (1.00, 3.00) | 2.00 (1.00, 3.00) | 2.00 (1.00, 4.00) | 0.034 |
| SOFA score | 2 (0.36) | 4 (0.72) | 9 (1.62) | 0.087 |
| SEPSIS, n (%) | 23 (4.13) | 21 (3.78) | 16 (2.87) | 0.509 |
| COPD, n (%) | 18 (3.23) | 20 (3.6) | 25 (4.49) | 0.527 |
| CHF, n (%) | 4 (0.72) | 12 (2.16) | 22 (3.95) | 0.001 |
| AMI, n (%) | 46 (8.35) | 81 (14.73) | 190 (34.36) | < 0.001 |
| DM, n (%) | 15 (2.69) | 30 (5.4) | 33 (5.92) | 0.023 |
| Pneumonia, n (%) | 102 (18.31) | 102 (18.35) | 75 (13.46) | 0.043 |
| Arrhythmia, n (%) | 106.23 ± 23.43 | 122.43 ± 31.84 | 172.34 ± 74.73 | < 0.001 |
| Glucose, mean (SD), mg/dL | 18.17 ± 12.42 | 18.27 ± 10.82 | 21.11 ± 14.51 | < 0.001 |
| BUN, median (IQR), mg/dL | 0.83 (0.69, 1.05) | 0.90 (0.73, 1.12) | 0.92 (0.76, 1.29) | < 0.001 |
| Serum creatinine, median (IQR), mg/dl | 145.50 ± 37.49 | 157.59 ± 40.53 | 174.83 ± 53.62 | < 0.001 |
| TC, median (IQR), mg/dL | 68.00  (55.00, 84.00) | 110.00  (91.00, 128.25) | 176.00  (138.00, 238.00) | < 0.001 |
| TG, median (IQR), mg/dL | 81.72 ± 31.88 | 92.08 ± 36.37 | 96.16 ± 44.71 | < 0.001 |
| LDL-C, mean (SD), mg/dL | 50.29 ± 16.27 | 43.18 ± 13.82 | 38.23 ± 13.33 | < 0.001 |
| HDL-C, mean (SD), mg/dL | 3.96 ± 0.50 | 3.93 ± 0.53 | 3.98 ± 0.57 | 0.261 |
| Serum, potassium, mean (SD), mmol/L | 139.00 ± 3.99 | 139.31 ± 3.35 | 138.99 ± 3.84 | 0.278 |
| Sodium, mean (SD), mmol/L | 8.15 ± 0.27 | 8.76 ± 0.16 | 9.59 ± 0.49 | < 0.001 |
| TyG, mean (SD) | 33 (5.92) | 53 (9.53) | 72 (12.93) | < 0.001 |
| Hospital 28 mortality, n (%) | 70.69 ± 15.24 | 69.17 ± 13.87 | 65.55 ± 12.72 | < 0.001 |

SOFA, sequential organ failure assessment; DM, diabetes mellitus; COPD, chronic obstructive pulmonary disease; CHF, congestive heart failure; AMI, acute myocardial infarction; BUN, blood urea nitrogen; TC, total cholesterol; TG, triglycerides; LDL-C, low-density lipoprotein cholesterol; HDL-C, high-density lipoprotein cholesterol; TyG: triglyceride–glucose.

SD, standard deviation; IQR, interquartile range.

| Table S6.  The univariate analysis of the baseline variables and 28-day hospital mortality | | |
| --- | --- | --- |
| **Variables** | **Hazard ratios (95% CI)** | **P value** |
| Age (years) |  |  |
| <60 | 1(Ref) |  |
| >=60 | 1.893 (1.233–2.905) | 0.004 |
| Gender |  |  |
| Male | 1(Ref) |  |
| Female | 1.091 (0.795–1.496) | 0.590 |
| Ethnicity |  |  |
| Caucasian | 1(Ref) |  |
| African American | 0.667 (0.4–1.111) | 0.120 |
| Others/Unkown | 0.981 (0.622–1.548) | 0.936 |
| Mechanical ventilation use |  |  |
| No | 1(Ref) |  |
| Yes | 3.037 (2.214–4.166) | <0.001 |
| SOFA score | 1.273 (1.201–1.348) | <0.001 |
| Diabetes mellitus |  |  |
| No | 1(Ref) |  |
| Yes | 1.262 (0.879–1.813) | 0.207 |
| COPD |  |  |
| No | 1(Ref) |  |
| Yes | 1.553 (0.84–2.868) | 0.160 |
| CHF |  |  |
| No | 1(Ref) |  |
| Yes | 0.844 (0.413–1.721) | 0.640 |
| AMI |  |  |
| No | 1(Ref) |  |
| Yes | 1.196 (0.56–2.555) | 0.644 |
| Pneumonia |  |  |
| No | 1(Ref) |  |
| Yes | 1.444 (0.896–2.329) | 0.131 |
| Arrhythmia |  |  |
| No | 1(Ref) |  |
| Yes | 1.398 (0.986–1.983) | 0.060 |
| Serum creatinine (mg/dL) Tertile |  |  |
| 0.24 - 0.9 | 1(Ref) |  |
| 0.91 - 16.1 | 1.528 (1.105–2.113) | 0.010 |
| BUN (mg/dL) Tertile |  |  |
| 4-16 | 1(Ref) |  |
| 17 - 144 | 1.758 (1.25–2.474) | 0.001 |
| Serum potassium (mmol/L) Tertile |  |  |
| 2.4 - 3.9 | 1(Ref) |  |
| 4.0 - 7.4 | 0.953 (0.693–1.31) | 0.767 |
| Serum sodium(mmol/L) Tertile |  |  |
| 116 - 139 | 1(Ref) |  |
| 140 - 160 | 1.24 (0.899–1.709) | 0.190 |
| CHG Tertile |  |  |
| Q1 (4.06-5.20) | 1(Ref) |  |
| Q2 (5.21-5.62) | 1.446 (0.93–2.247) | 0.101 |
| Q3 (5.63-7.92) | 1.707 (1.125–2.592) | 0.012 |
| TyG Tertile |  |  |
| Q1 (6.72-8.49) | 1(Ref) |  |
| Q2 (8.5-9.04) | 1.49 (0.964–2.302) | 0.072 |
| Q3 (9.04-12.33) | 1.832 (1.212–2.77) | 0.004 |
| SOFA score, sequential organ failure assessment score; COPD, chronic obstructive pulmonary disease; CHF, congestive heart failure; AMI, acute myocardial infarction; BUN, Blood urea nitrogen; CHG: cholesterol, high-density lipoprotein, and glucose index; TyG: triglyceride–glucose. | | |

| Table S7. Cox regression models for the association between the CHG index, TyG index, and 28-day in-hospital mortality after excluding individuals with missing values. | | | | | | | |
| --- | --- | --- | --- | --- | --- | --- | --- |
| **Categories** | **Event, (n%)** | **Model1** | | **Model2** | | **Model3** | |
|  |  | **HR (95% CI)** | **P value** | **HR (95% CI)** | **P value** | **HR (95% CI)** | **P value** |
| **CHG index** | | | | | | | |
| Continuous | 157 (9.5) | 1.581 (1.218–2.052) | <0.001 | 1.818 (1.381–2.393) | <0.001 | 1.601 (1.184–2.164) | 0.002 |
| Quartile | | | | | | | |
| Q1 | 32 (5.8) | 1(Ref) |  | 1(Ref) |  | 1(Ref) |  |
| Q2 | 51 (9.3) | 1.435 (0.922–2.234) | 0.110 | 1.515 (0.971–2.365) | 0.067 | 1.425 (0.901–2.253) | 0.130 |
| Q3 | 74 (13.5) | 1.726 (1.137–2.62) | 0.010 | 1.977 (1.291–3.028) | 0.002 | 1.758 (1.12–2.759) | 0.014 |
| P for trend |  |  | 0.011 |  | 0.002 |  | 0.014 |
| **TyG index** | | | | | | | |
| Continuous | 157 (9.5) | 1.456 (1.189–1.783) | <0.001 | 1.666 (1.34–2.07) | <0.001 | 1.433 (1.118–1.836) | 0.005 |
| Quartile | | | | | | | |
| Q1 | 33 (6) | 1(Ref) |  | 1(Ref) |  | 1(Ref) |  |
| Q2 | 52 (9.5) | 1.47 (0.95–2.275) | 0.084 | 1.525 (0.985–2.362) | 0.059 | 1(Ref) |  |
| Q3 | 72 (13.1) | 1.833 (1.213–2.771) | 0.004 | 2.125 (1.391–3.246) | <0.001 | 1.469 (0.942–2.291) | 0.09 |
| P for trend |  |  | 0.004 |  | <0.001 |  | 0.054 |

Model 1: unadjusted

Model 2: adjusted for age, gender, and ethnicity

Model 3: adjusted for Model 2 plus, ventilation status, SOFA score, diabetes, sepsis, COPD, CHF, AMI, arrhythmia, pneumonia, serum creatinine, BUN, serum potassium, and sodium levels

CHG: cholesterol, high-density lipoprotein, and glucose index; TyG: triglyceride–glucose.

| Table S8. Cox regression models for the association between the CHG index, TyG index, and 28-day in-hospital mortality after multiple imputations. | | | | | | | |
| --- | --- | --- | --- | --- | --- | --- | --- |
| **Categories** | **Event, (n%)** | **Model1** | | **Model2** | | **Model3** | |
|  |  | **HR (95% CI)** | **P value** | **HR (95% CI)** | **P value** | **HR (95% CI)** | **P value** |
| **CHG index** | | | | | | | |
| Continuous | 158 (9.5) | 1.554 (1.198–2.018) | <0.001 | 1.762 (1.34–2.317) | <0.001 | 1.559 (1.156–2.104) | 0.004 |
| Quartile | | | | | | | |
| Q1 | 32 (5.7) | 1(Ref) |  | 1(Ref) |  | 1(Ref) |  |
| Q2 | 52 (9.4) | 1.446 (0.93–2.247) | 0.101 | 1.518 (0.974–2.364) | 0.065 | 1.423 (0.903–2.243) | 0.129 |
| Q3 | 74 (13.3) | 1.707 (1.125–2.592) | 0.012 | 1.931 (1.261–2.955) | 0.003 | 1.728 (1.102–2.71) | 0.017 |
| P for trend |  |  | 0.013 |  | 0.002 |  | 0.018 |
| **TyG index** | | | | | | | |
| Continuous | 158 (9.5) | 1.436 (1.175–1.755) | <0.001 | 1.611 (1.303–1.992) | <0.001 | 1.395 (1.096–1.775) | 0.007 |
| Quartile | | | | | | | |
| Q1 | 33 (5.9) | 1(Ref) |  | 1(Ref) |  | 1(Ref) |  |
| Q2 | 53 (9.5) | 1.49 (0.964–2.302) | 0.072 | 1.54 (0.996–2.381) | 0.052 | 1.48 (0.951–2.304) | 0.082 |
| Q3 | 72 (12.9) | 1.832 (1.212–2.77) | 0.004 | 2.099 (1.375–3.205) | <0.001 | 1.552 (0.986–2.445) | 0.058 |
| P for trend |  |  | 0.004 |  | <0.001 |  | 0.069 |

Model 1: unadjusted

Model 2: adjusted for age, gender, and ethnicity

Model 3: adjusted for Model 2 plus, ventilation status, SOFA score, diabetes, sepsis, COPD, CHF, AMI, arrhythmia, pneumonia, serum creatinine, BUN, serum potassium, and sodium levels

CHG: cholesterol, high-density lipoprotein, and glucose index; TyG: triglyceride–glucose.


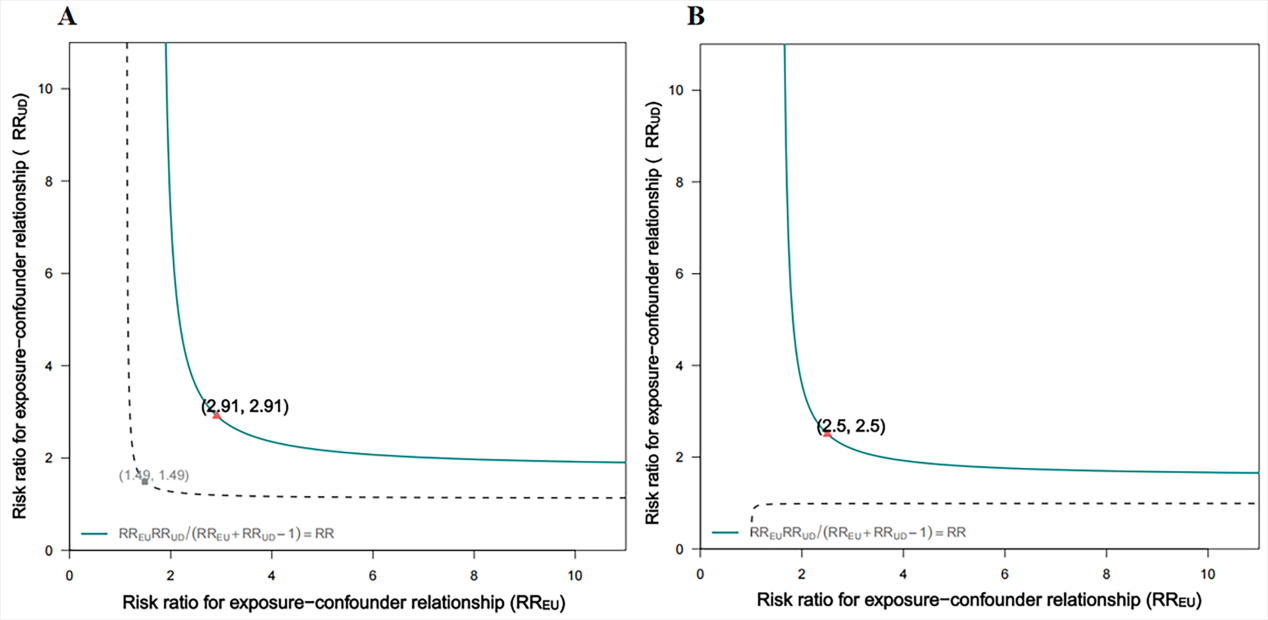


Figure S1 E-value plot assessing the risk ratios of the CHG index and TyG index in relation to 28-day in-hospital mortality. Adjusted for age, gender, ethnicity, ventilation status, SOFA score, diabetes, sepsis, COPD, CHF, AMI, arrhythmia, pneumonia, serum creatinine, BUN, serum potassium, and sodium levels.
